# CTNNBIP1-CLSTN1 Functions as a Housekeeping Chimeric RNA, and Regulates Cell Proliferation through SERPINE2

**DOI:** 10.21203/rs.3.rs-3112431/v1

**Published:** 2023-07-17

**Authors:** Hui Li, Chen Chen, Yue Tang, Fujun Qin, Sandeep Singh

**Affiliations:** School of Basic Medical Sciences; Zhengzhou University; Zhengzhou University; University of Virginia

## Abstract

The conventional wisdom that chimeric RNAs being peculiarity of carcinoma, and the products of chromosomal rearrangement is being challenged, However, experimental evidence supporting chimeric RNAs in normal physiology being functional is scarce. We decided to focus on one particular chimeric RNA, *CTNNBIP1-CLSTN1*. We examined its expression among various tissues and cell types, and compared quantitatively among cancer and non-cancer cells. We further investigated its role in a panel of non-cancer cells and probed the functional mechanism. We found that this fusion transcript is expressed in almost all tissues, and a wide range of cell types including fibroblasts, epithelial, stem, vascular endothelial cells, and hepatocytes. The expression level in non-cancerous cell lines is also not evidently different from that in the cancer cell lines. Furthermore, silencing *CTNNBIP1-CLSTN1* significantly reduces cell proliferation rate, by inducing G2/M arrest in cell cycle progress and apoptosis in at least three cell types. Importantly, rescue experiments confirmed that the cell cycle arrest can be regained by exogenous expression of the chimera, but not the wild type parental gene. Further evidence is provided that *CTNNBIP1-CLSTN1* regulates cell proliferation through *SERPINE2*. Thus, *CTNNBIP1-CLSTN1* represents an example of a new class of fusion RNA, dubbed “housekeeping chimeric RNAs”.

## 1. BACKGROUND

The discovery of the *BCR-ABL* fusion gene by Nowell et al. (1) during 1960s, as a result of chromosomal translocation in chronic myelogenous leukemia, has provided a strategy to those who are in search of gene fusions involved in various neoplasm(2, 3). Even since then, the prevailing view is that gene fusions are cancer specific, and they then make fusion products (RNAs and proteins), which can be also used as specific biomarkers and drug targets (4, 5). This assumption has resulted in the explosion of Mitelman Database of Chromosome Aberrations and Gene Fusions in Cancer in the Cancer Genome Anatomy Project. However, this very dogma has been challenged, when more and more studies and researches led to the continuous finding of chimeric RNAs in the normal physiology, especially with the availability of a large number of transcriptome sequencing (RNA-seq) databases (6). The prevalence of chimeric RNA has so far exceeds our expectations (7–9), with several characteristic modalities(7, 10), and, at least some of them also play key roles in various settings (7, 8, 11, 12). In particular, a group of chimeric RNAs are suggested to serve a housekeeping role, in that their expression are generally ubiquitous, and are indispensible for cell survival and maintenance.

We previously implemented and analyzed approximately 300 RNA sequencing libraries established from 30 different non-neoplastic human tissues and cell lines, which led to the discovery of 9,778 chimeric RNAs that are potential products of cis-splicing of adjacent genes (cis-SAGe) or RNA trans-splicing (7). In that study, the great majority of the chimeric RNAs are tissue-specific, and around 10% occur in more than one sample, of which 51 in more than five different samples. Among these recurrent chimeras, *CTNNBIP1-CLSTN1* was found in a large number of tissues, and seemed to be indispensible for the culture of an immortalized astrocyte cell line (7). In addition, we demonstrated that it is a cis-SAGe chimeric RNA(13), composed of two neighboring parental genes located on chr.1p36 (7, 13), and induced when transcription factor CTCF is silenced (9). However, the exact role the chimeric RNA plays, and the mechanism of its functionality are not clear.

We hypothesize the existence of a group of chimeric RNAs functioning as “housekeeping” chimeric RNAs, i.e., playing an universal role in maintaining physiological functions in various non-cancer human cell types. In this study, we aim to investigate whether *CTNNBIP1-CLSTN1* chimeric RNA represents a member of this new class of chimeric RNAs, and to further investigate its downstream pathway. We first confirmed the presence of this transcript in different types of tissues and cells, and from these, three different cell lines were further selected to study the role of the chimera related to cell proliferation, migration and apoptosis. Transcriptome sequencing and downstream analyses were further conducted to uncover its functional mechanism.

## 2. METHODS

### 2.1 Cell culture, siRNA and transfections

HEK-293T, HUVEC, and LO2 cells were maintained in DMEM/HIGH GLUCOSE medium with 4500 mg/L Glucose and 4.0 mM L-Glutamine (HyClone^™^, USA), supplemented with 10% fetal bovine serum (LONSERA, Uruguay), and 1% penicillin and streptomycin (HyClone^™^, USA). The cells were incubated in 37°C, 5% CO_2_, with medium changes every other day. Cells were digested by 0.25% trypsin with 1g/L EDTA (HyClone^™^, USA). siRNAs were synthesized by Sangon Biotech (Shanghai, China) and transfected with Lipofectamine RNAiMAX (Life Technologies, USA) following the manufacture’s protocols. Transfection efficiencies were evaluated 48 hours after siRNA transfection.

The targeting sequences are:

si-Negative Control, CGTACGCGGAATACTTCGA;

siCTNNBIP1, GGAAGAGTCCGGAGGAGAT;

siCTNNBIP1-CLSTN1, TGCTTGTTAACCTGGTCGA.

### 2.2 RNA extraction, quantitative reverse transcription PCR (qRT-PCR) and sanger sequencing

RNA was extracted from cells with BEI-BEI BIOTECH Total RNA Isolation Kit (Zhengzhou, China) and reverse-transcribed by TIANGEN FastKing cDNA Kit (Beijing, China) according to the manufacture’s instructions. Primers used for the fusion have been described earlier (13). qRT-PCR was carried out using the ABI StepOne Plus Real-Time PCR system (Life Technologies) with TB Green^®^ Premix Ex Taq^™^ (TaKaRa, Japan), and followed by gel electrophoresis with Gel-Red^™^ Nucleic Acid Gel Stain (Biotium, California, USA). Data were analyzed using ΔΔCt statistical method, with the expressions was normalized against internal control GAPDH. Axygen^®^ AxyPrep DNA Gel Extraction Kit (USA) was used for DNA purification, and followed by Sanger sequencing validation at Sangon Biotech.

The primer sequences for qRT-PCR are:

WT-CTNNBIP1:F:5’-CTCATGCTGCGGAAGATGGGAT-3’; R:5’-CTGGAAAACGCCATCACCACGT-3’;

FN1: F:5’-CCACCCAATGTTCAGCTCAC-3’; R:5’-GTAGCATCTGTCACACGAGC-3’;

SERPINE2: F:5’-CTTCCTCTTGGCCTCTGTGA-3’; R:5’-ACGCCGTATCTCATCACCAT-3’

### 2.3 Vector construction of *CTNNBIP1-CLSTN1* and plasmid transfection

The full length cDNA of *CTNNBIP1-CLSTN1*, and the wild type *CTNNBIP1* open reading frame were cloned into pCDNA3.1 plasmid vector system and verified by Sanger sequencing. For transient expression, the plasmids were transfected into cells with Lipofectamine 2000 (Life Technologies, USA) following the manufacture’s instruction.

### 2.4 Cell proliferation assay

HEK-293T, HUVEC and LO2 were seeded on 96-well plates with appropriate confluence. 72 hours after siRNAs or plasmids transfections, cells proliferation was measured with DOJINDO Cell Counting Kit-8 (Japan) and incubated at 37°C, 5% CO_2_ for two hours. Cell viability in each well was determined by O.D. value at 450 nm.

### 2.5 Rescue experiment

For CCK8 assay, HEK-293T cells were seeded in 96-well plates with 2,000 per well. Cells were first transfected with pCDNA3.1 plasmids expressing *CTNNBIP1-CLSTN1, CTNNBIP1, SERPINE2, FN1* or empty control respectively, all plasmids were synthesized by Genscript and confirmed by Sanger sequencing; 24 hours later, the cells were transfected with siCTNNBIP1-CLSTN1, siCTNNBIP1 or NC; 48 hours after siRNA transfection, 10 μl CCK8 was added in each well and incubated for two hours at 37°C, 5% CO_2_. Cell viability in each well was determined by O.D. value at 450 nm. In live cell imaging, HEK-293T were seeded and transfected the same way as cell proliferation assay, then the plates were placed under a live-cell-imaging microscope (JULI Stage, NanoEnTek, Seoul, South Korea) after siRNA transfection for 60 hours. Images were captured every six hours for measurement of cell proliferation. A total of 10 cycles were captured. Image analysis was performed for cell proliferation analysis using JULI Stat (NanoEnTek, Seoul, South Korea), and a cell growth curve was plotted according to the cell growth density.

### 2.6 Wound healing assay

HEK-293T, HUVEC and LO2 cells were seeded in 6-well plates and transfected with siRNAs or plasmids with proper reagents. Around 48 hours after transfections, when cell confluence reached more than 90%, a scratch was made by manual scratching longitudinally on the bottom of each well with a 10 μl plastic pipette tip. The plates were rinsed twice with PBS, and replaced with fresh DMEM complete media. After that, the plates were placed under a live-cell-imaging microscope (JULI Stage, NanoEnTek, Seoul, South Korea) and incubated at 37°C, 5% CO_2_. Images were captured every two hours and the gap distance after the scratch was documented, with a total of 12 cycles captured. Picture analysis was performed by Wound healing analysis using JULI Stat (NanoEnTek, Seoul, South Korea). A wound density curve according to the percentage of cell migration measured as the recovered wound area relative to the original wound area was plotted.

### 2.7 Flow cytometry

72 hours after siRNA transfections, HEK-293T, HUVEC and LO2 cells were digested with 0.25% trypsin and washed twice with PBS, and cell density was adjusted to 1×10^6^ cells/ml. Next, the cells were fixed with pre-cooled 70% alcohol overnight at 4°C, washed twice with PBS, and treated with 500 μl propidium iodide (PI) (KeyGEN BioTECH, Nanjing, China) reaction mixture containing RNase under dark conditions for 60 minutes at room temperature. Cell cycle stages were detected by flow cytometer (FACS Calibur, Becton, Dickinson and Company, New Jersey, USA) and analyzed by Flow Jo software. For cell apoptosis detection, the cells were collected and washed as above, then mixed with 500 μl binding buffer containing 5 μl Annexin V-FITC and 5 μl PI stain (KeyGEN BioTECH, Nanjing, China), reacted in dark place for 15 minutes at room temperature. The flow cytometer was applied for detecting cell apoptosis and analyzed by Flow Jo.

### 2.8 Cell fractionation

HEK-293T, HUVEC and LO2 cells were digested with 0.25% trypsin, washed once with PBS, then separated into two fractions using NE-PER nuclear and cytoplasmic extraction reagents (Thermo Fisher, USA) following the manufacturer’s instructions. RNA from each part was extracted respectively with BEI-BEI BIOTECH Total RNA Isolation Kit, followed by qRT-PCR with *MALAT1* and *GAPDH* as controls.

## 3. RESULTS

### 3.1 *CTNNBIP1-CLSTN1* is widely expressed among human tissues and cell types.

**Figure 1:**
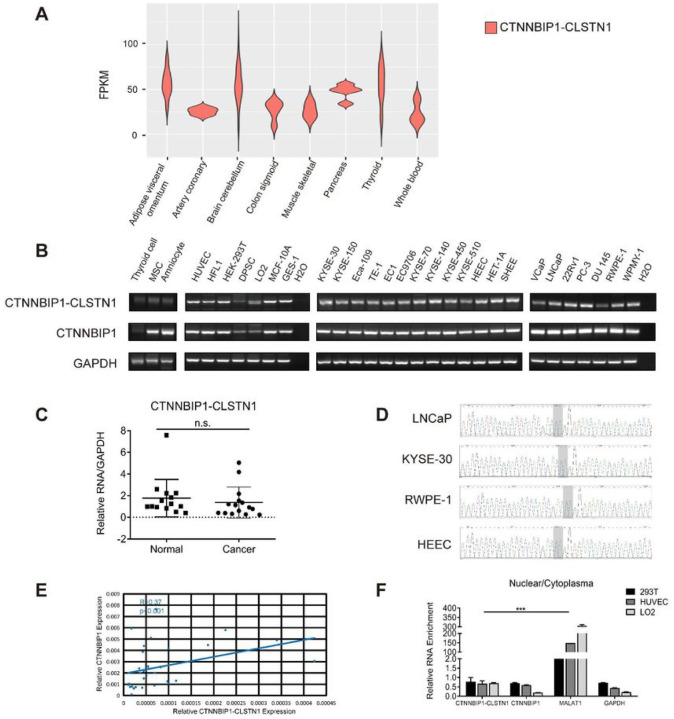
The expression of CTNNBIP1-CLSTN1 in various human tissues and cell lines. **(A)** The expression of *CTNNBIP1-CLSTN1* in whole Genotype-Tissue Expression (GTEx). FPKM of the chimeric RNA in various tissues were plotted. **(B)** RT-PCR detecting the expression of *CTNNBIP1-CLSTN1* in several human cell lines, followed by gel-electrophoresis. Internal control, *GAPDH*, and wild type parental gene *CTNNBIP1* were also included. **(C)** The expression level of the chimera was measured in 15 non-cancer cell lines and 15 cancer cell lines using qRT-PCR. The expression of the fusion was normalized against internal control, *GAPDH*. **(D)** Sanger sequencing of RT-PCR products in cancer cell lines, LNCaP, KYSE-30, and non-cancer cell lines, RWPE-1, HEEC. Light gray area marks the fusion junction site. **(E)** Correlation between the relative expression level of fusion and *CTNNBIP1* was plotted (R=0.37, P<0.001). **(F)** HEK-293T, HUVEC and LO2 cells were fractioned into nuclear and cytoplasmic parts. Expression of the chimera and *CTNNBIP1* in each part were measured by qRT-PCR. *GAPDH* and known long non-coding RNA *MALAT1* were used as controls. Ratios of expression in nuclear and cytoplasm parts were plotted. Asterisks indicate statistical significance: *P < 0.05. **P < 0.01.***P < 0.001.

Previously, we identified 291 recurrent chimeric RNAs by analyzing RN-Seq data from around 300 non-cancer tissues and cells (7). *CTNNBIP1-CLSTN1* is one of them, detected by RT-PCR in liver, lung, kidney etc. We recently expanded our chimeric RNA search to the whole Genotype-Tissue Expression (GTEx), which contains 9,495 non-diseased human tissue samples from 53 different tissues (14, 15). From this study, we noticed that *CTNNBIP1-CLSTN1* was detected in almost all samples (Fig. 1A and Fig. S1).

We then performed RT-PCR to detect the fusion and its parental genes in 15 non-cancer cell lines belonging to 12 different tissue of origins, including cell types of fibrocytes, amniocytes, epithelial cells, stem cells, and vascular endothelial cells, followed by gel-electrophoresis (Fig. 1B). *CTNNBIP1-CLSTN1* was discovered in all the samples. Using qRT-PCR, we quantified its expression in 15 non-cancer cells, and 15 cancer cell lines of esophageal and prostate cancer. There was no statistically significant difference in the expression level between cancer and normal lines (Fig. 1C, and Fig. S2-S3). The junction sequence was shown exact the same among different cancer cell lines, LNCaP and KYSE-30, and non-cancer lines, RWPE-1 and HEEC by Sanger sequencing (Fig. 1D). We also monitored the wild type parental transcripts using primers covering at least one exon not shared with the fusion transcript. Even though the wild type *CTNNBIP1* transcript was readily detectable, the wild type *CLSTN1* was not. We also found that the relative expressions of the chimeric fusion and *CTNNBIP1* are positively correlated (Pearson’s correlation R = 0.37, P < 0.001) (Fig. 1E, and Fig. S2-S3), consistent with the chimeric RNA being a product of 5’ gene read-through, and suggesting that the great majority of *CLSTN1* transcripts is used to form chimeric RNA.

This chimeric RNA is predicted to encode an in-frame chimeric protein. Here, we decided to take advantage of the fact that regular protein-coding mRNAs are mainly present in the cytoplasm, while long-non-coding RNAs often reside in the nucleus to regulate transcription (16–18). We chose human embryonic kidney cell HEK-293T, human umbilical vein endothelial cell HUVEC, and hepatocyte line LO2 cells for fractionation assay and extracted RNA to detect the chimeric RNA by qRT-PCR. Classic protein-coding gene *GAPDH* and long non-coding RNA *MALAT1* were used as controls. Not surprisingly, *MALAT1* was found mostly in the nuclear fraction. On the contrary, *CTNNBIP1-CLSTN1*, wild type *CTNNBIP1* and *GAPDH* were all enriched in the cytoplasmic part, (Fig. 1F). This observation is consistent with the previous experiment of Western blot (7), further supporting that the fusion has a protein-coding function.

### 3.2. Silencing *CTNNBIP1-CLSTN1* reduced cell proliferation and cell motility

**Figure 2:**
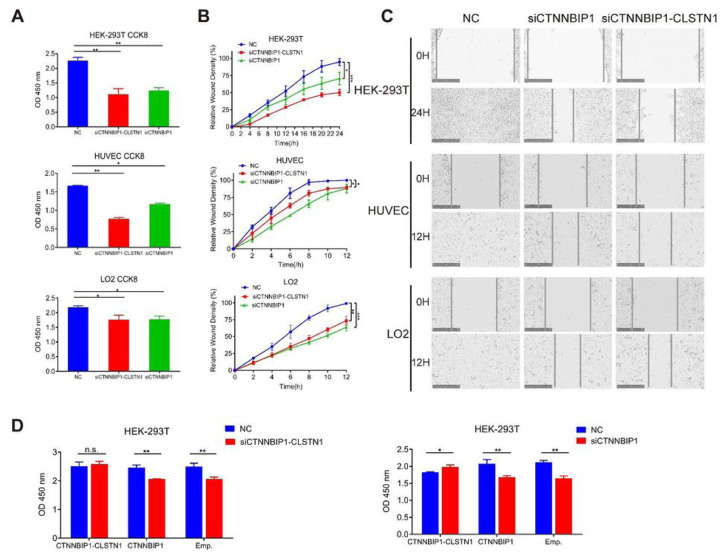
Knocking down *CTNNBIP1-CLSTN1* results in significance reduction in cell proliferation and cell motility in HEK-293T, HUVEC and LO2 cell lines. **(A)** CCK8 assay was used to measure the cell proliferation rate 72 hours after siRNA transfection. Both siRNAs resulted in significant cell growth suppression in three cell lines. **(B)** Both siRNAs resulted in significant reduction of cell motility in three cell lines. **(C)** Representative microscopy images of cells transfected NC, siCTNNBIP1-CLSTN1 and siCTNNBIP1 showed movement across a wound at zero (upper panel) and 12 hours (lower panel) in HUVEC and LO2, zero (upper panel) and 24 hours (lower panel) in HEK-293T. Scale bars represent 250 μm. **(D)** In a rescue experiment, HEK-293T were transfected with plasmid expressing *CTNNBIP1-CLSTN1*, *CTNNBIP1* or empty control, and 24 hours later, the cells were transfected with siCTNNBIP1-CLSTN1 or siCTNNBIP1 and negative control (NC). Cell viability was measured by CCK8 after 48 additional hours. Asterisks indicate statistical significance: *P < 0.05. **P < 0.01.***P < 0.001.

Based on its ubiquitous expression pattern, we hypothesized that the chimeric RNA may belong to the group of chimeric RNAs, which we call “housekeeping chimeric RNAs”. Indeed, previous report has documented its indispensible role in immortalized astrocytes (7). To further investigate the function of *CTNNBIP1-CLSTN1*, and support its basic role of principle cell maintenance, we transfected siRNAs targeting the chimera in multiple human non-cancer cell lines, HEK-293T, HUVEC and LO2 cell lines. The siRNA, siCTNNBIP1-CLSTN1 targeting the fusion junction sequence resulted in significant reduction of the fusion transcript, but not the wild-type *CTNNBIP1* in all three cell lines (Fig. S4 and S5). Since, we could not design a siRNA that specifically silences the wild type CTNNBIP1, as a control, we designed a siRNA, siCTNNBIP1 that targets the common region sequence in the fusion and wild type *CTNNBIP1*. As predicted, this siRNA lead to the silencing of both the fusion and wild type *CTNNBIP1* (Fig. S5). Both siCTNNBIP1-CLSTN1 and siCTNNBIP1 significantly decreased cell proliferation based on CCK8 measurement (Fig. 2A). Cell migration was monitored by wound healing assay with live-cell-imaging microscopy after scratching. The percent relative wound density was calculated by measuring the density of cells that migrated into the original wound. Both siCTNNBIP1-CLSTN1 and siCTNNBIP1 significantly inhibited cell migration compared to the negative control (Fig. 2B). Figure 2C shows representative microscopic images of cells across a wound at zero and 12 hours in HUVEC and LO2 cells, and zero and 24 hours in HEK-293T cells. These results suggest that *CTNNBIP1-CLSTN1* plays a significant role in general cell growth and movement, regardless of cell types.

To rule out the off-target effect of siRNA, we performed rescue experiments by transfecting in the construct of CTNNBIP1-CLSTN1 expressing plasmid and construct encoding the wild type *CTNNBIP1*. The reduced cell proliferation was indeed rescued by the *CTNNBIP1-CLSTN1* construct in both siCTNNBIP1-CLSTN1 and siCTNNBIP1 groups. In contrast, the wild type *CTNNBIP1* failed to rescue in either groups (Fig. 2D), suggesting the effect of reduced cell proliferation was caused by the fusion silencing, but the wild type *CTNNBIP1*.

### 3.3. Silencing *CTNNBIP1-CLSTN1* resulted in cell cycle arrest and apoptosis

**Figure 3:**
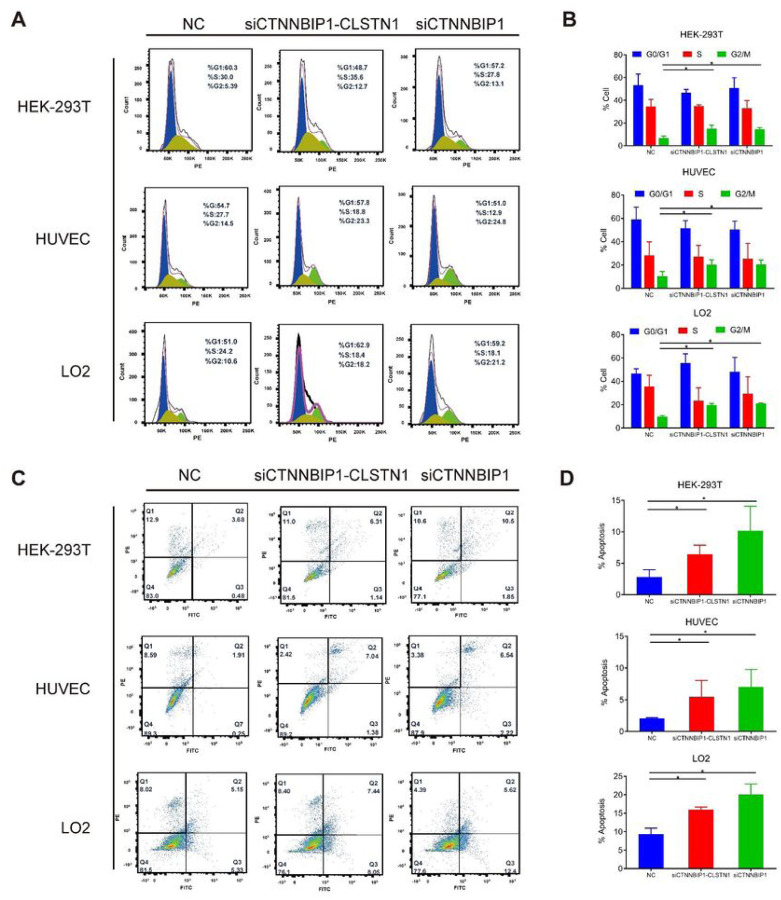
Knocking down *CTNNBIP1-CLSTN1* resulted in G2/M cell cycle arrest and apoptosis. **(A)** Cell cycle stages 72 hours after siRNAs transfection were examined by flow cytometry and analyzed by FlowJo. Representative pictures were shown. **(B)** Silencing *CTNNBIP1-CLSTN1* induced G2/M arrest. **(C)** Cell apoptosis was examined at 72 hours after siRNAs transfection by flow cytometry and analyzed by FlowJo. Representative pictures were shown. **(D)** Silencing *CTNNBIP1-CLSTN1* induced apoptosis. Data were presented as mean±SD. Asterisks indicate statistical significance: *P < 0.05.

To investigate the mechanism of the reduced cell proliferation caused by silencing the fusion, we performed flow cytometry to evaluate cell cycle with propidium iodide staining after siRNA transfection. Cell numbers in G2/M phase were notably increased in both siRNA groups that silenced the fusion, compared to that in the NC group (Fig. 3A). This G2/M arrest was observed in all three cell lines (Fig. 3B).

We then evaluated cell apoptosis using Annexin V-FITC and propidium iodide staining. The results from the flow cytometry assay showed that siCTNNBIP1 and siCTNNBIP1-CLSTN1 transfection noticeably increased apoptosis in all three cell lines, compared to the control (Fig. 3C and Fig. 3D).

### 3.4. Overexpression of *CTNNBIP1-CLSTN1*, but not the wild type *CTNNBIP1*, promotes cell growth and cell migration

**Figure 4:**
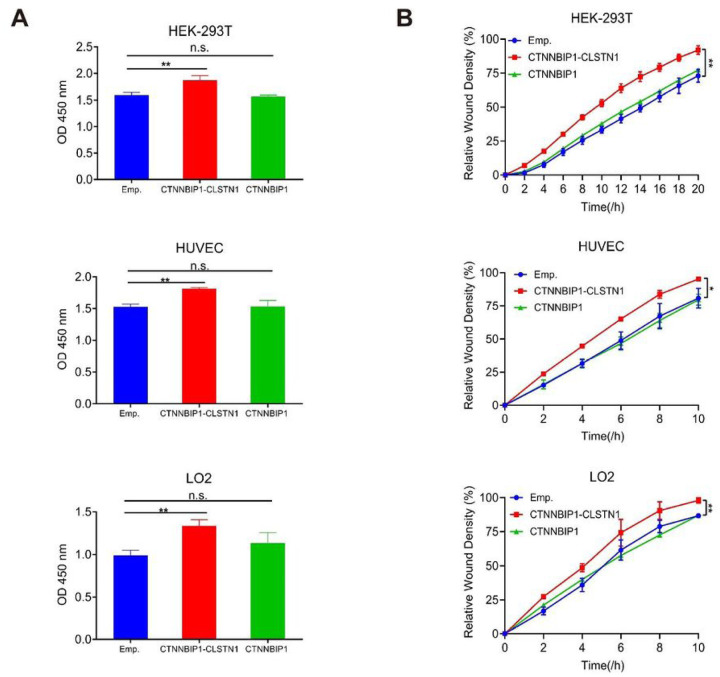
Overexpression of CTNNBIP1-CLSTN1, but not wild type CTNNBIP1, promotes cell proliferation and cell motility. **(A)** HEK-293T, HUVEC and LO2 were transfected with pCDNA3.1 plasmids transcribing full-length coding regions of *CTNNBIP1-CLSTN1* (CTNNBIP1-CLSTN1), *CTNNBIP1* (CTNNBIP1) or empty vector (Emp.). CCK8 was used to measure the cell proliferation rate 72 hours after plasmid transfection. Overexpression of the fusion promoted cell proliferation rate, whereas overexpression of the wild type CTNNBIP1 had no such effect. **(B)** Overexpression of the fusion also promoted cell motility, whereas overexpression of the wild type *CTNNBIP1* had no such effect.

In this experiment, HEK-293T, HUVEC, and LO2 cells were transfected with either pCDNA3.1 plasmid that encodes the full-length CDS of *CTNNBIP1-CLSTN1*, or *CTNNBIP1*, or the empty vector respectively. In contrast to the loss-of-function experiments, CCK8 assay showed that the expression of the chimera, but not the wild type *CTNNBIP1*, promoted cell proliferation in all three cell lines (Fig. 4A). Consistently, wound healing assay also demonstrated that the overexpression of *CTNNBIP1-CLSTN1*, but not *CTNNBIP1* enhanced the cell migration ability in all three cell lines (Fig. 4B).

### 3.5. *CTNNBIP1-CLSTN1* influences cell proliferation by regulating autocrine signaling factors *SERPINE2*

**Figure 5:**
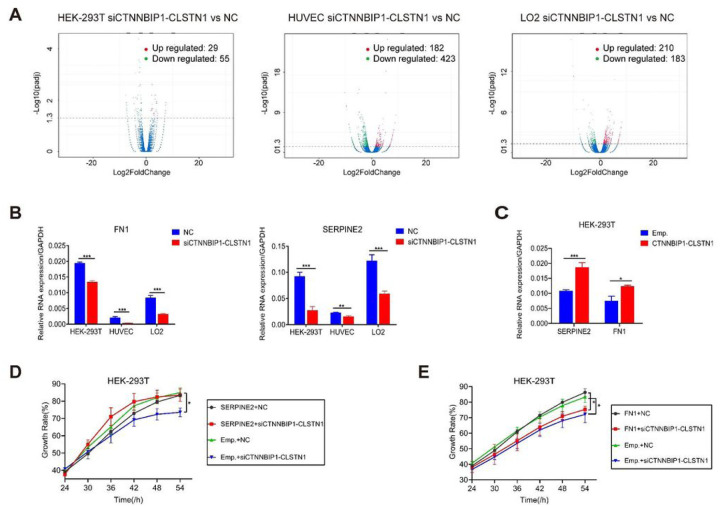
*CTNNBIP1-CLSTN1* regulates cell proliferation through *SERPINE2*. **(A)** Volcano plots of DEGs in HEK-293T, HUVEC, and LO2. Red dots indicate significantly up-regulated genes. Green dots indicate significantly down-regulated genes. Blue dots indicate no significant difference (P value<0.05). **(B)** qRT-PCR detecting expression of *FN1* and *SERPINE2* at 48 hours after siRNAs transfection. Silencing *CTNNBIP1-CLSTN1* significantly suppressed *FN1* and *SERPINE2*. **(C)** Overexpression of *CTNNBIP1-CLSTN1* increased the expression of *FN1* and *SERPINE2*. **(D)** In a rescue experiment, the reduced cell growth rate caused by siCTNNBIP1-CLSTN1 was recovered by transfecting the cells with *SERPINE2* expression plasmid. **(E)** Overexpression of *FN1* had no such rescue effect. The horizontal axis represents the time after plasmid transfection. Asterisks indicate statistical significance: *P < 0.05. **P < 0.01.***P < 0.001.

To investigate the downstream pathway mediating the effect of *CTNNBIP1-CLSTN1* on cell proliferation, we performed transcriptome sequencing analysis of HEK-293T, HUVEC and LO2 cells transfected with siCTNNBIP1-CLSTN1 and negative control siRNA. Differential expression analysis between the two groups in three cell lines was conducted, obtaining 84, 605, and 393 differentially expressed genes (DEGs) (Supplementary Table 1), respectively. Volcano plot of DEGs in three cell lines are shown in Fig. 5A (padj < 0.05). Gene Ontology (GO) terms for DEGs were also examined. 135 enriched GO terms in HEK-293T, 286 enriched GO terms in HUVEC, and 137 enriched GO terms in LO2 were found (Supplementary Table 1). Several terms showed unconformity in different cells, and were consistent with the general role the fusion plays. However, there are also specific GO terms unique to individual cell lines, suggesting that the fusion may have additional roles that are cell type specific. The top 13 for HEK-293T, the top 23 for HUVEC, and the top 14 for LO2 in the GO term list are displayed (Fig. S6).

Out of the DEGs, Fibronectin 1 (*FN1*) and Serine protease inhibitor E2 (*SERPINE2*) stand out, owing to the fact that they both were down-regulated in siCTNNBIP1-CLSTN1 group of all three cell lines. Furthermore, they were included in several GO terms related to cell growth (GO:0001558, GO:0040008, and GO:0016049). Fibronectin 1(*FN1*)(19, 20), is a member of the glycoprotein family that is widely expressed by multiple cell types (GTEx data, Fig. S7A). Serine protease inhibitor E member 2 (*SERPINE2*) (21, 22), also called Protease Nexin-1(*PN-1*) belongs to the Serpin gene super family, is also ubiquitously expressed in human tissues and cells (GTEx, Fig. S7B). We first confirmed their expression by qRT-PCR (Fig. 5B). Both were reduced upon the silencing of the fusion. In contrast, overexpression of *CTNNBIP1-CLSTN1* up-regulated both genes (Fig. 5C). We then investigated whether the effect of the fusion is mediated by these two genes. To do so, we transfected in expression plasmids encoding *FN1* or *SERPINE2* in cells with siCTNNBIP1-CLSTN1 or siCT. As indicated in Fig. 5D, SERPINE2 can restore the cell proliferation rate to a normal level. In contrast, *FN1* failed to rescue the reduced cellular growth induced by siCTNNBIP1-CLSTN1 (Fig. 5E), suggesting that the effect of the chimeric RNA on cell proliferation is mostly mediated by SERPINE2.

## 4. DISCUSSION

In recent years, exploration of gene fusions, and chimeric RNAs in various carcinomas has progressed by leaps and bounds (23). Numerous examples were found by high throughput approaches, including microarray and next generation sequencing, for instance, *TMPRSS2-ETS* (24, 25) and *D2HGDH-GAL3ST2* (26) in prostate cancer, *LHX6-NDUFA8* and *SLC2A11-MIF* in cervical cancer (27), *GOLM1-MAK10* in esophageal squamous cell carcinoma (ESCC) (28), *EML4-ALK* in non-small cell lung (NSCLC) (29), *CHFR-GOLGA3* in Bladder cancer (30), *RRM2-C2orf48* in colorectal cancer (CRC) (31), and ASTN2-PAPPA_as_ in esophageal cancer (32). Most of these gene fusions are significantly over-expressed in cancer, compared to non-cancer tissues and cells. In addition, some of them were verified to have clinical correlations between their expression level and cancer stage and/or patient survival (26). Without doubt, they represent effective markers for clinical diagnosis/prognosis, and/or drug targets.

However, in the recent years, continuous discoveries have demonstrated that chimeric RNA is not a unique phenomenon to cancer, nor is it all due to the composition of gene fusion. It is widespread in normal human tissues and cells (7, 8, 33–36). *JAZF1-JJAZ1* is observed in endometrial stromal cells, and instead of chromosome rearrangement, it is derived from RNA trans-splicing there (37). Similarly *DUS4L-BCAP29*, which is a product of cis-splicing of adjacent genes exists not only in prostate cancer and gastric cancer as previously reported (38, 39), but is also present in various normal tissues (40). In a previous study, 291 fusion transcripts were found by analyzing nearly 300 RNA-Seq libraries, with *CTNNBIP1-CLSTN1* being observed in five non-neoplastic tissues (7). In another study, Singh et al. explored the landscape of chimeric RNAs in 9,495 Genotype-Tissue Expression (GTEx) samples and established a dataset containing a total of 7,193 chimeric RNAs (8), where *CTNNBIP1-CLSTN1* being detected in the majority of samples. The results we presented here provide further evidences that CTNNBIP1-CLSTN1 is commonly expressed in miscellaneous cell types, and to a similar level in normal and cancer cells. Furthermore, this fusion also plays basic physiological roles that sustain cell growth, and cell migration. These evidences support the classification of this fusion as a member of housekeeping chimeric RNAs.

**Figure 6:**
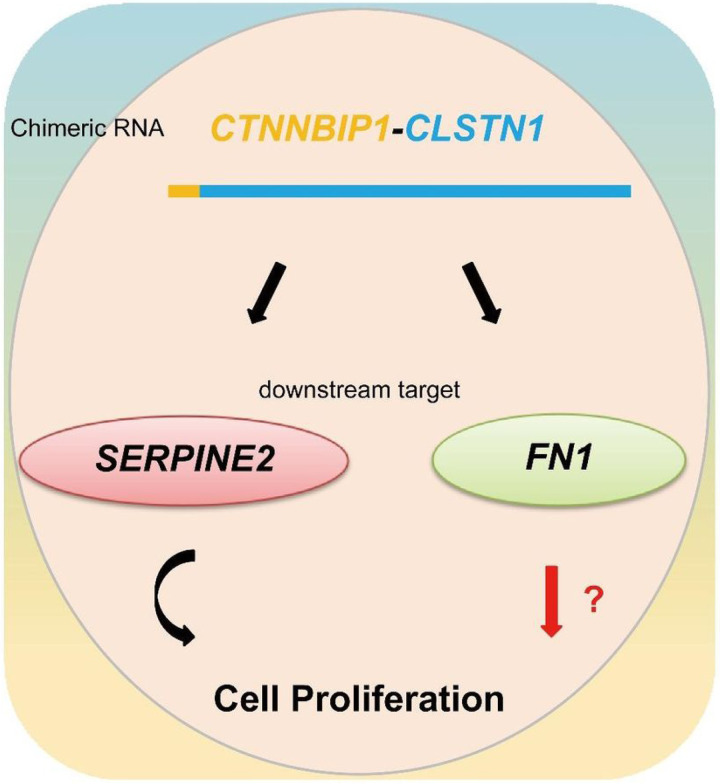
An illustration of chimeric RNA *CTNNBIP1-CLSTN1, and its functional mechanism*. *CTNNBIP1-CLSTN1* regulates the expression of two downstream target genes, *SERPINE2* and *FN1*. Silencing the fusion reduces cell proliferation rate, which can be rescued by *SERPINE2*, but not *FN1*. It is possible that *FN1* mediates other cell physiological roles of the fusion.

Transcriptome sequencing exploration connects the fusion with downstream targets including *FN1*, and *SERPINE2*. *FN1* is an adhesive glycoprotein of the extracellular matrix with a variety of binding domains for cell surface and extracellular ligands (19, 20), and involved in some biological processes including cell vitality and apoptosis through these multiple interaction sites (41, 42). *SERPINE2* is an extracellular plasminogen activator inhibitor with the effect of inhibiting various protease activities (21, 22), and function in many physiological processes including inflammation, cell growth and metastasis (43, 44). They are both ubiquitously expressed in human tissues and cells (Fig. S7). Rescue experiments confirmed that the expression of *SERPINE2* can restore the cell proliferation to a normal level, suggesting its role in mediating the effect of *CTNNBIP1-CLSTN1* on cell proliferation. However, the expression of *FN1* failed to have any rescue effect on cell proliferation, suggesting that it may play other roles (Fig. 6).

Besides the common DEGs among the three different cell models, there are groups of differentially expressed genes that are found in only two or one cell models, suggesting that the fusion may also have cell-specific roles. The exact mechanism of how the fusion RNA regulates the expression of its downstream genes remains unclear, and will be the directions of our future studies.

## CONCLUSION

In summary, *CTNNBIP1-CLSTN1* is ubiquitously expressed in normal and cancer cells. It regulates cell proliferation in various cell types, and thus represents a new class of RNA, housekeeping chimeric RNA. We believe more of such chimeric RNAs exist. Their presence challenges the traditional dogma that chimeric fusion RNAs are cancer specific, and sounds the alarm for the practice of rushing fusion RNAs discovered from cancer tissue/cells into biomarkers and/or therapeutic targets.

## Data Availability

The Raw and processed RNA-Sequencing data from this study have been submitted to the NCBI Gene Expression Omnibus (GEO; http://www.ncbi.nlm.nih.gov/geo/) under accession number GSE165479. The security token for reviewers to access the data before it is publicly available is gjqlgkamtxgltgn. Flow cytometry data has been deposited into Flow Repository. The ID and URL with secret code for reviewers are listed below: Cell cycle: FR-FCM-Z3E3 http://flowrepository.org/id/RvFrm5qG1S7akXGqSKb85uWhbRTZ0ACn5uCIPoGiulAygdG2Lq0SnQRYez7L5oHB Cell apoptosis: FR-FCM-Z3E4 http://flowrepository.org/id/RvFrzD1pnAaXP44GVUKW0wVl1QimdHNOKR1hXyc07ao0xIZODy0tzrEgOykrnMUg All the other data supporting the findings of this study are available within the article and its supplementary information files and from the corresponding author upon reasonable request. A reporting summary for this article is available as a Supplementary Information file.

## LIST OF ABBREVIATIONS

cis-SAGe: GTEx, ESCC, NSCLC, CRC
